# Asthma control in primary care: the results of an observational cross-sectional study in Italy and Spain

**DOI:** 10.1186/s40413-017-0144-5

**Published:** 2017-04-06

**Authors:** Maria Sandra Magnoni, Manuela Latorre, Germano Bettoncelli, M. Guadalupe Sanchez-Herrero, Araceli Lopez, Eduardo Calvo, Andrea Rizzi, Marco Caminati, Gianenrico Senna, Pierluigi Paggiaro

**Affiliations:** 1 Medical and Scientific Department, GlaxoSmithKline, Via A. Fleming 2, 37135 Verona, Italy; 2 Cardio-Thoracic and Vascular Department, University of Pisa, Pisa, Italy; 3 Società Italiana di Medicina Generale, Scuola Europea di Medicina Generale, Firenze, Italy; 4 Medical Department, GlaxoSmithKline, Tres Cantos Madrid, Spain; 5 Centro de Salud Universitario Pozuelo Estación, Madrid, Spain; 6 Allergy Unit, Verona University Hospital, Verona, Italy

**Keywords:** Asthma, Control, ACT

## Abstract

**Background:**

Poor asthma control observed in several surveys may be related to a lack of systematic assessment by physicians and/or to patient underestimation of symptoms. Along this line, the purpose of this study was to investigate the level of asthma control in patients attending the GP office for different reasons, either for renewal of drug prescription or for worsening of asthma symptoms.

**Methods:**

Each of the 145 General Practitioners (GP) in Italy and Spain selected at least eight asthmatic patients attending their office for a renewal of drug prescription (Group A) or for worsening of asthma symptoms (Group B), between May and December 2009. Asthma Control Test (ACT) and other clinical information (including SF-12 questionnaire) were collected.

**Results:**

Data from 1375 patients with moderate-severe asthma were analysed (mean age: 47.2 years; female: 59%; smokers or ex-smokers: 35.4%); 57% were on treatment with ICS-LABA combination. ACT score < 20 (uncontrolled asthma) was observed in 77.8% Group B patients, as expected, but also in 28.6% Group A patients. Uncontrolled patients reported their asthma being well or fairly well controlled in 68.4% of cases. Risk factors for uncontrolled asthma were older age, asthma severity, and smoking habit. In uncontrolled patients, GPs changed or increased the level of therapy in 75.8% and initiated asthma treatment in 61.3% of cases, in association with educational intervention, closer monitoring or pulmonologist consultations.

**Discussion:**

The systematic use of ACT in asthmatics attending GP’s clinic may detect high rates of uncontrolled patients who underestimate their clinical conditions, particularly those asking solely for asthma medication renewal. Poor adherence to daily drug therapy was reported in more than 40% of patients and could be an important contributor of uncontrolled asthma.

**Conclusions:**

The results highlight the importance of routine longitudinal assessment of asthma patients in primary care and point to the need for an increased attention to asthma management by GPs.

## Background

Although Global Initiative for Asthma (GINA) provides guidelines for optimal management of asthma [1] in order to reach and maintain good control of the disease, several observational studies show that the majority of asthmatic patients are not controlled. The Asthma in Reality in Europe (AIRE) study, based on telephone interviews to a large group of patients, showed that only 5.3% of the surveyed population fulfilled all criteria of asthma control as indicated by the GINA guidelines; furthermore, approximately 50% of patients considered their asthma to be completely or well controlled despite reporting severe persistent symptoms [2], thus showing large discrepancy between patients’ perception of control and actual severity of the disease. More recently, an internet-based survey confirmed that asthmatic patients tend to overestimate their asthma control, with 55% of them reporting several limitations in daily life activities despite frequent visits to General Practitioners (GPs) [3]. Finally, a large study performed in primary care settings reported that asthma control in Italy was not optimal, with many patients requiring unscheduled GP visits, Emergency Room attendance and hospitalization because of asthma [4].

The reasons why reaching good asthma control is still so difficult are various, including inappropriate pharmacologic treatment and poor adherence to the asthma management plan. The latter may be related to the attitude of many patients to underestimate symptom severity and frequency, refusing regular therapy when symptoms are mild or absent. These considerations suggest that greater attention to the level of asthma control should be paid by both patients and physicians, possibly with the help of simple and accurate tools. For this reason the Asthma Control Test (ACT) has been developed and validated. This simple 5-item questionnaire allows computing a single score which reflects the symptom control domain of asthma control. This tool may increase the patient’s awareness of asthma control, allowing to monitor the outcome of the disease and helping the patients to understand when a consultation with the physician is needed [5, 6]. In combination with spirometry and Peak Expiratory Flow (PEF) monitoring, ACT may represent an important tool for GPs for monitoring and adjusting pharmacologic treatment.

In clinical practice, asthmatic patients usually consult their GPs for two main reasons: asthma deterioration (in order to review their current therapy) or prescription renewal regardless of asthma control. Checking the level of asthma control in seemingly stable patients, attending the GP clinic only for prescription renewal, may be a good opportunity to evaluate asthma control, health-related quality of life and health resource utilization.

The ACTIS study (**A**sthma **C**ontrol **T**est study in **I**taly and **S**pain) was performed in two European countries (Italy and Spain) with the main objective of evaluating the level of asthma control in a sample of asthmatic patients spontaneously attending the GP clinic for different reasons. The hypothesis was to find out whether patients referring to GPs for a simple renewal of drug prescription had actually poor asthma control. In these patients GPs usually confirm the previous treatment, with no further assessment of their asthma; in these cases, ACT evaluation may provide useful information for home-based management, allowing early detection of asthma deterioration or poor asthma control not properly perceived by the patients. Furthermore, we evaluate the relationship between asthma control and risk factors as well as the mental and physical components of the health status.

## Methods

ACTIS (GSK study 111595) is a multicenter and multinational (Italy and Spain) observational cross-sectional study, performed in GP’s offices.

The study was approved by the Ethic Committees and all the patients provided written informed consent.

The primary objective was the evaluation of the prevalence of uncontrolled asthma (defined as an ACT score < 20) in two different populations: a) patients attending the GP’s clinic only for prescription renewal of currently used anti-asthmatic drugs (Group A); b) patients attending the GP’s clinic for asthma symptom worsening (Group B). Secondary objectives were the assessment of health-related quality of life (by SF-12 questionnaire), consumption of disease-related healthcare resources in the previous 6 months (in particular, loss of working days, GP’s or pulmonary specialist’s visits, Emergency Room attendance or hospitalization for asthma), adherence to therapy.

### Study design

One hundred forty five GPs in Italy and Spain voluntarily agreed to participate to the study, which was performed from May 2009 to December 2009. Each GP was to recruit at least eight patients with known diagnosis of asthma, consecutively attending their clinic for one of these two reasons: 1) renewal of drug prescription (Group A, at least 4 patients per GP); 2) recent worsening of asthma symptoms requiring a change in current treatment (Group B, at least 4 patients per GP).

Inclusion criteria were: male or female with age ≥ 18 years, asthma diagnosed at least 6 months before the study (with or without having performed pulmonary function tests), ability to understand and fill the questionnaires used in the study, and willing to sign the informed consent for the participation to the study and the use of personal data. Exclusion criteria were mainly the presence of pulmonary diseases other than asthma (like COPD or bronchiectasias) or other severe cardiovascular or metabolic diseases.

Patients were administered by GPs: a) Asthma Control Test (ACT); b) SF-12 (only to the Italian sample of patients), a shortened and validated version of the SF-36 questionnaire, exploring health-related quality of life by computing two indices, one related to physical status and one related to mental status; a higher score indicates a better physical or mental status [7]; c) a 4-item questionnaire, called Morinsky-Green (validated and well known), aimed at investigating patient’s compliance with treatment, specifically if patients stop taking their medicines when they feel better (only in the Spanish sample of patients).

Each GP filled an electronic record form, which included information on patient clinical history, asthma severity, current therapy, healthcare resource consumption in the last 6 months (GP’s or pulmonary specialist’s visits, Emergency Room attendance, hospitalizations for asthma), as well as general data (age, gender, smoking habit, etc.).

GPs then evaluated the results of ACT and assessed whether current therapy was adequate to the level of asthma control. Treatment was consequently modified and the new prescription was recorded.

The protocol was approved by local Ethic Committees and written informed consent was obtained from each patient before the inclusion into the study.

### Statistical analysis

The analyses performed were mainly descriptive: mean, standard deviation, median, range and quartiles were reported for numerical variables; absolute values and percentages were computed for qualitative variables. Missing values were excluded for both numerical and qualitative variables.

Univariate logistic analyses have been performed to study the relationship between some independent variables (such as age, gender, asthma severity, duration of the disease) and ACT score as dependent variable. Analysis of variance (ANOVA) was performed to evaluate differences between groups for the above mentioned independent variables, for markers of health resource consumption (loss of work days, number of visits to GP, etc.) and of quality of life as assessed by SF-12 questionnaire.

## Results

The ACTIS study involved 145 GPs. Overall, 1375 patients were recruited: 56% attended the GP’s clinic for renewal of drug prescription (Group A) and 44% for worsening of asthma symptoms (Group B).

### Main characteristics of the enrolled patients

Table 1 shows patient demographic and clinical findings: 40.7% were male, mean age 47.2 years. Mean age at the onset of asthma was about 30 years: in 26.8% of cases asthma begun in childhood or adolescence, whereas in 7.2% after 60 years. The clinical diagnosis of asthma was confirmed by functional tests (spirometry with/without methacholine challenge) in 56.1% of the subjects, and the time span since the last spirometry was of about 2 years in both groups. The majority of patients were on inhaled corticosteroid/long-acting beta2-agonist (ICS/LABA) combinations. There was no significant difference between patients from Group A and patients from Group B with regard to anthropometric findings, general characteristics of the disease (duration of asthma, performance of spirometry) and type of pharmacologic treatment. Also, the characteristics of the recruited subjects in Italy and Spain were similar.

**Table 1:** Demographics and main characteristics of the recruited asthmatic patients

		Reasons for GP consultation	
	Total	A: Asking for asthma drugs prescription renewal	B: seeking for medical advice due to symptom worsening
Total (n)	1375	777	598
Male (n, %)	560 (40.7)	313 (40.3)	247 (41.3)
Female (n, %)	815 (59.3)	464 (59.7)	351 (58.7)
Age (mean, SD)	47.2 (17.6)	46.0 (17.7)	48.7 (17.4)
Smoking habits			
• non smoker	888 (64.6)	522 (67.2)	366 (61.2)
• smoker	265 (19.3)	130 (16.7)	135 (22.6)
• ex smoker	222 (16,1)	125 (16,1)	97 (16.2)
Mean duration of asthma (years, SD)	12.8 (10.1)	12.6 (9.9)	13.0 (10.4)
Clinical diagnosis (n, %)	604 (43.9)	349 (44.9)	255 (42.6)
Spirometry test (n, %)	771 (56.1)	428 (55.1)	343 (57.4)
Time to last spirometry (years, SD)	2.0 (2.7)	2.2 (3.0)	1.8 (2,2)
Therapy			
- Short-acting beta2-agonists (SABA)	814(59.2)	458(58.9)	356(59.5)
- Long-acting beta2-agonists (LABA)	242(17.6)	131(16.9)	111 (18.6)
- Inhaled Corticosteroids (ICS)	397(28.9)	206(26.5)	191(31.9)
- Antihistamines	414(30.1)	230 (29.6)	184 (30.8)
- Leukotriene modifiers	272(19.8)	142 (18.3)	130 (21.7)
- LABA/ICS combinations	793 (57.7)	454(58.4)	339 (56.7)
- Omalizumab	5 (0.4)	3 (0.4)	2 (0.3)
- Systemic corticosteroids	187(13.6)	86 (11.1)	101(16.9)
- Methylxantines	58 (4.2)	21(2.7)	37 (6.2)
- Anticholinergic agents	128 (9.3)	54 (6.9)	74(12.4)
Other(phytodrugs, homeopathicagents,ecc)	20 (1.5)	7 (0.9)	13 (2.2)

### The control of asthma using ACT

**Fig. 1:**
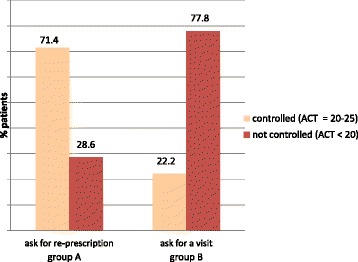
Subjects with uncontrolled asthma (ACT < 20) in Group A (patients asking for asthma drug prescription renewal) and Group B (patients with symptoms worsening)

Subjects with uncontrolled asthma (ACT < 20) were about 50% of the whole study population. As expected, in Group B (patients with symptom worsening) the percentage of uncontrolled asthma was high (77.8%); however, even in Group A (patients asking for renewal of drug prescription) 28.6% of patients showed uncontrolled asthma. The difference between the two groups was statistically significant (*p* < 0.0001 by chi-square test) (Fig. 1).

When asked about perception of their level of asthma control, patients with uncontrolled asthma reported to be well or fully controlled in 20% of cases, and partially controlled in 48% of cases. Only a minority of these patients (31.4%) believed that their asthma was poorly controlled. In the univariate analysis, lack of control significantly correlated with age (Odds Ratio, OR = 1.216), asthma severity (OR = 3.413), smoking habit (smokers vs ex- and non-smokers: OR = 1.474), duration of asthma (OR = 1.092) and country (Italy vs Spain OR = 1.469) (Table 2). Furthermore, Group B patients reported a higher rate of loss of working days, consultations to GP, visits to ER or hospitalizations than Group A patients (data not shown).

**Table 2:** Relation between poor asthma control and independent variables (univariate logistic regression); reference group was patients with ACT ≥ 20

	OR	95%CI	*P* value
Age (10 year groups)	1.216	1.144 - 1.294	<0.0001
Asthma severity (mild/moderate/severe)	3.413	2.815 - 4.139	<0.0001
Asthma duration (5 years groups)	1.092	1.036 - 1.153	0.0013
Smoke (smokers vs no/ex)	1.474	1.124 - 1.932	0.0049
Country (Italy vs Spain)	1.469	1.187 - 1.818	0.0004
Gender (male vs female)	1.024	0.891 - 1.234	n.s.

### Quality of life measured by the SF-12 questionnaire

The SF-12 questionnaire was administered to the Italian sample of patients. The physical component score derived from the SF-12 questionnaire was lower in patients from Group B than in patients from Group A (40.5 vs 46.8, *p* < 0.001) and in patients with uncontrolled asthma than in those with ACT score ≥ 20 (39.6 and 49.2 respectively, *p* < 0.001). Same trends for Group B vs Group A (43.5 vs 48.1, *p* < 0.001) and for uncontrolled vs controlled asthmatics (43.4 vs 49.1 respectively, *p* < 0.001) were observed for the mental component score. In a multivariate analysis, both physical and mental component scores were greater (indicating a better quality of life) in patients with good or total asthma control, in younger patients, in those with less severe asthma and in those with shorter duration of the disease (the latter only for the physical component).

### Compliance to drug therapy

**Fig. 2:**
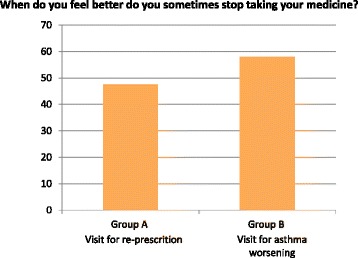
Patients who abandon the treatment when they feel better

There is no difference in the two groups in the percentage of patients that at times forget to take their medications (39.9% in group A and 47.3% in group B). However, the number of patients who abandon the treatment when they feel better is significantly higher in patients from Group B than in patients from Group A: 47.6% vs 58% (Chi-square 0.012) (Fig. 2).

### Therapeutic decisions of GPs after the assessment of control

**Fig. 3:**
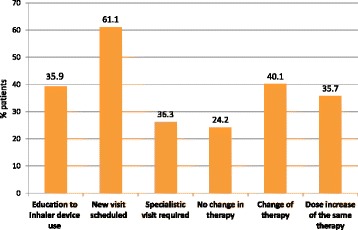
Actions undertaken by GPs in patients with poor asthma control (ACT ≤ 19)

Table 3 and Fig. 3 report the actions undertaken by GPs when presented with a patient who had poor asthma control. In 40.1% of cases GPs changed the level of therapy and in 35.7% increased the dose of the current medication. As a whole, 63% of poorly controlled patients, not on a controller therapy at the moment of recruitment, were prescribed an anti-asthmatic treatment, either as a first prescription or as a renewed prescription (data not shown); therapy was changed or increased in 75.8% of uncontrolled patients already on treatment. In 61.1% of cases GPs scheduled a new visit, whereas pulmonary/allergology specialist visits or hospitalizations were prescribed in 26.2 and 0.7% of patients respectively (mostly in Group B). An educational intervention (mainly on the use of inhalers) was performed in 38% of patients. As a whole, interventions undertaken by GPs were slightly more frequent in Group B than in Group A.

**Table 3:** GP’s actions in poorly controlled asthmatic patients (ACT < 20)

	Total		Reasons for GP consultation			
			A: asking for asthma drugs prescription renewal		B: seeking for medical advice due to symptom worsening	
	N	%	N	%	N	%
N. patients (n.d. = 1)	686	100	222	100.0	464	100.0
No change in therapy	166	24.2	98	44.1	68	14.7
Dose increase of the same therapy	245	35.7	76	34.2	169	36.4
Change of therapy	275	40.1	48	21.6	227	48.9
Other actions (N. patients = 687)						
None	88	12.8	40	18.0	48	10.3
New visit scheduled	420	61.1	119	53.6	301	64.7
Specialistic visit required:	180	26.2	44	19.8	136	29.2
pneumologist	146	21.3	37	16.7	109	23.4
allergologist	33	4.8	8	3.6	25	5.4
other	2	0.3	.	.	2	0.4
Emergency visit/hospitalization	5	0.7	1	0.5	4	0.9
Laboratory test	175	25.5	42	18.9	133	28.6
Education to inhaler device use	269	39.2	88	39.6	181	38.9
Other	24	3.5	8	3.6	16	3.4

In 14.7% of patients from Group B and in 44% of patients from Group A, GPs maintained the same level of therapy despite an inadequate control of asthma. More than 50% of uncontrolled patients in Group A increased their treatment levels because of poor ACT score.

## Discussion

The results of the ACTIS study show that the level of asthma control in Italy and Spain, as assessed by ACT in patients attending the GP’s clinic, is still poor. Although some bias in the selection of the patients might be considered, almost 50% of the selected patients were still uncontrolled. Nonetheless, the level of control in these two countries, as well as in other European countries, seems to be increased in the present study with respect to the AIRE study, performed almost 10 years earlier [2]. This may be due to several reasons, including a larger availability of effective anti-asthmatic medications related to the implementation of asthma guidelines, as demonstrated in some countries [8], and to an increased awareness of the disease in both physicians and patients [9]. However, asthma control is still far from being optimal and further improvement is desirable.

Poor adherence to daily drug therapy is recognized as an important contributor to deterioration of asthma control, with increased morbidity and mortality [10]. Real life studies report high rates of discontinuation of controller treatments in asthmatic patients [11, 12]. The reasons for non-adherence are varied and are likely to include insufficient inhaler technique, the complexity of the therapeutic regimens (e.g. multiple daily dosing) as well as patient’s beliefs about therapy, such as fear of adverse effects or the perception that medication should be used in response to symptoms more than on a regular basis [10, 13]. In keeping with the latter observation, in our study a high percentage (>40%) of patients discontinued the treatment when they felt better, suggesting a “symptoms, no asthma” belief. Not surprisingly, treatment discontinuation was significantly higher in group B (patients with symptom worsening).

The most interesting finding in this study is that the percentage of uncontrolled asthma is high not only in patients attending GP’s clinic for worsening of asthma symptoms, but also in patients attending GP’s clinic for a simple renewal of drug prescriptions (77.8 and 28.6%, respectively). While it is not surprising that almost 80% of Group B patients were uncontrolled, the novel observation is that almost 30% of Group A patients were uncontrolled according to ACT. The reason why these patients requested only a renewal of drug prescription and not a reassessment of their asthma might be due to a sort of “adaptation” to the constant presence of symptoms and/or limitations in daily life, which are considered inevitable and not susceptible of improvement. The observation that more than half of uncontrolled patients perceive their asthma as “under control” is in favor of this hypothesis. Thus, in agreement with previous studies [2, 3], patients largely overestimate their asthma control level, thus precluding a more effective management of the disease; the wrong conviction that “normal life” cannot be attained may lead asthmatic patients to tolerate uncontrolled symptoms without asking the GP or the pulmonary specialist for medical advice, in order to improve asthma management.

An important practical implication of these data is that GPs should systematically evaluate asthma control in all patients attending their clinic, even in those asking a simple prescription renewal. Physician should also educate patients about the possibility of improving asthma control with currently available drugs and therapeutic strategies: this could lead to a better quality of life and a lower consumption of socio-economic resources, as the use of rescue medication, unscheduled visit to GPs or pulmonary specialists, emergency department access or hospitalization.

Patients included in this study are a good sample of asthmatics well known to GPs: they are middle-aged, mainly female, with high percentages of current or ex-smokers. The data disagree with the high prevalence of mild asthmatics found in population-based epidemiological studies [14] and reflect greater asthma severity in patients attending the GP’s clinic. However, a limit of our study is the non-random selection of the patients, which may have led to inclusion of more severe patients. Also, the high rate of intervention by GPS in patients who were uncontrolled by ACT cannot be compared with a control group, not included in the study design.

Our results are in keeping with those of previous studies [15], showing that older age, asthma severity and duration as well as smoking habits are associated with poor asthma control. Increasing evidence suggests that the efficacy of glucocorticoids is reduced in smoking-asthmatics [16] and it has been reported that a high proportion of patients admitted to the emergency department for asthmatic exacerbation consists of smokers [17].

The finding of poor asthma control prompted GPs to modify therapy in 75.8% of patients and to perform an educational intervention in 39.2% of patients. On the other hand, in a consistent proportion of uncontrolled patients, GPs did not increase the level of anti-asthma therapy. The reason of a lack of a “step-up” approach in these cases is not clear. However, GINA Guidelines recommend an accurate assessment of the adherence and of the inhalation technique before stepping-up [1], and this might have been occurred also in this case. Furthermore, in uncontrolled patients, GPs scheduled a more stringent monitoring or required a pulmonologist consultation. Thus, the extensive use of ACT may lead GPs to a more active intervention to improve asthma management, particularly in patients asking only for drug prescription renewal. In these patients, a more accurate assessment of asthma control by using ACT may detect poor adherence to therapy, poor inhaling technique, inadequate level of pharmacologic treatment, or persistent exposure to asthma triggers.

## Conclusions

The systematic use of ACT in asthmatic patients attending the GP’s clinic may detect high rates of uncontrolled patients who underestimate their clinical conditions. Inadequate control might partly be due to underestimation of asthma symptoms and was associated with well known risk factors (older age, asthma severity, smoking habit). The recognition of poor asthma control induced physicians to increase intervention in asthma care. Therefore, a broader use of a simple and validated tool like ACT may help GPs to implement of asthma guidelines in clinical practice, in order to improve disease management and possibly reduce asthma morbidity.

## Abbreviations

ACT: Asthma Control Test
ACTIS: Asthma Control Test Study in Italy and Spain
AIRE study: Asthma in Reality in Europe study
GINA: Global Initiative for Asthma
GP: General Practitioner
ICS: Inhaled Corticosteroid
LABA: Long-acting beta2-agonist
PEF: Peak Expiratory Flow
SF-12 questionnaire: Short Form-12 questionnaire

## References

[1] Global Initiative for Asthma (GINA). Global strategy for asthma management and prevention: NHLBI/WHO Workshop report. Bethesda: National Institutes of Health, National Heart, Lung and Blood Institute; up date 2009

[2] Rabe KF, Vermeire PA, Soriano JB, Maier WC (2000). Clinical management of asthma in 1999: the Asthma Insights and Reality in Europe (AIRE) study. Eur Respir J.

[3] Demoly P, Paggiaro PL, Plaza V (2009). Prevalence of asthma control among adults in France, Germany, Italy, Spain and the UK. Eur Respir Rev.

[4] Caramori G, Bettoncelli G, Carone M (2007). Degree of control of physician-diagnosed asthma and COPD in Italy. Mon Arch Chest Dis.

[5] Nathan RA, Sorkness CA, Kosinski M (2004). Development of the Asthma Control Test: a survey for assessing asthma control. J Allergy Clin Immunol.

[6] LeNoir M, Williamson A, Stanford RH, Stempel DA (2006). Assessment of asthma control in a general population of asthmatics. Curr Med Res Opin.

[7] Ware J, Kosinsky M, Keller SD (1996). A 12-Item Short-Form Health Survey: construction of scales and preliminary tests of reliability and validity. Med Care.

[8] Haahtela T, Tuomisto LE, Pietinalho A (2006). A 10 year asthma program in Finland: major change for the better. Thorax.

[9] Braido F, Comaschi M, Valle I (2012). Knowledge and health care resource allocation: CME/CPD course guidelines-based efficacy. Eur Ann Allergy Clin Immunol.

[10] Ulrik CS, Backer V, Søes-Petersen U (2006). The Patient’s Perspective: Adherence or Non-adherence to Asthma Controller Therapy?. J Asthma.

[11] Breekveldt-Postma NS, Gerrits CM, Lammers JW (2004). Persistence with inhaled corticosteroid therapy in daily practice. Respir Med.

[12] Breekveldt-Postma NS, Koerselman J, Erkens JA (2008). Treatment with inhaled corticosteroids in asthma is too often discontinued. Pharmacoepidemiol Drug Saf.

[13] Pelaez S, Lamontagne AJ, Collin J (2015). Patients’perspective of barriers and facilitators to talking long term controller medication for asthma: a novel taxonomy. BMC Pulm Med.

[14] Cazzoletti L, Marcon A, Corsico A (2010). Asthma severity according to Global Initiative for Asthma and its determinants: an international study. Int Arch Allergy Immunol.

[15] De Marco R, Bugiani M, Cazzoletti L (2003). The control of asthma in Italy. A multicentre descriptive study on young adults with doctor diagnosed current asthma. Allergy.

[16] Chaudhuri R, Livingston E, McMahon AD (2003). Cigarettes smoking impairs the terapeutics response of oral corticosteroids in chronic asthma. Am J Respir Crit Care Med.

[17] Silverman RA, Boudreaux ED, Woodruff PC (2003). Cigarette smoking among the asthmatic adults presenting to 64 emergency departments. Chest.

